# Assessing HLA imputation accuracy in a West African population

**DOI:** 10.1371/journal.pone.0291437

**Published:** 2023-09-28

**Authors:** Ruth Nanjala, Mamana Mbiyavanga, Suhaila Hashim, Santie de Villiers, Nicola Mulder

**Affiliations:** 1 Department of Biochemistry and Biotechnology, Pwani University, Kilifi, Kenya; 2 Computational Biology Division, Department of Integrative Biomedical Sciences, IDM, University of Cape Town, Cape Town, South Africa; 3 Pwani University Biosciences Research Centre, Pwani University, Kilifi, Kenya; Biotechnology Perspectives Organization, SUDAN

## Abstract

The Human Leukocyte Antigen (HLA) region plays an important role in autoimmune and infectious diseases. HLA is a highly polymorphic region and thus difficult to impute. We, therefore, sought to evaluate HLA imputation accuracy, specifically in a West African population, since they are understudied and are known to harbor high genetic diversity. The study sets were selected from 315 Gambian individuals within the Gambian Genome Variation Project (GGVP) Whole Genome Sequence datasets. Two different arrays, Illumina Omni 2.5 and Human Hereditary and Health in Africa (H3Africa), were assessed for the appropriateness of their markers, and these were used to test several imputation panels and tools. The reference panels were chosen from the 1000 Genomes (1kg-All), 1000 Genomes African (1kg-Afr), 1000 Genomes Gambian (1kg-Gwd), H3Africa, and the HLA Multi-ethnic datasets. HLA-A, HLA-B, and HLA-C alleles were imputed using HIBAG, SNP2HLA, CookHLA, and Minimac4, and concordance rate was used as an assessment metric. The best performing tool was found to be HIBAG, with a concordance rate of 0.84, while the best performing reference panel was the H3Africa panel, with a concordance rate of 0.62. Minimac4 (0.75) was shown to increase HLA-B allele imputation accuracy compared to HIBAG (0.71), SNP2HLA (0.51) and CookHLA (0.17). The H3Africa and Illumina Omni 2.5 array performances were comparable, showing that genotyping arrays have less influence on HLA imputation in West African populations. The findings show that using a larger population-specific reference panel and the HIBAG tool improves the accuracy of HLA imputation in a West African population.

## Introduction

The Major Histocompatibility Complex (MHC) region is a large locus in the human genome composed of polymorphic Human Leukocyte Antigen (HLA) genes. The MHC region, found on the short arm of chromosome 6, spans around 5Mbp and contains over 200 genes, with 128 predicted to be expressed [1]. It is one of the most complex regions in the human genome due to the high density of polymorphism and linkage disequilibrium [2].

**Fig 1:**
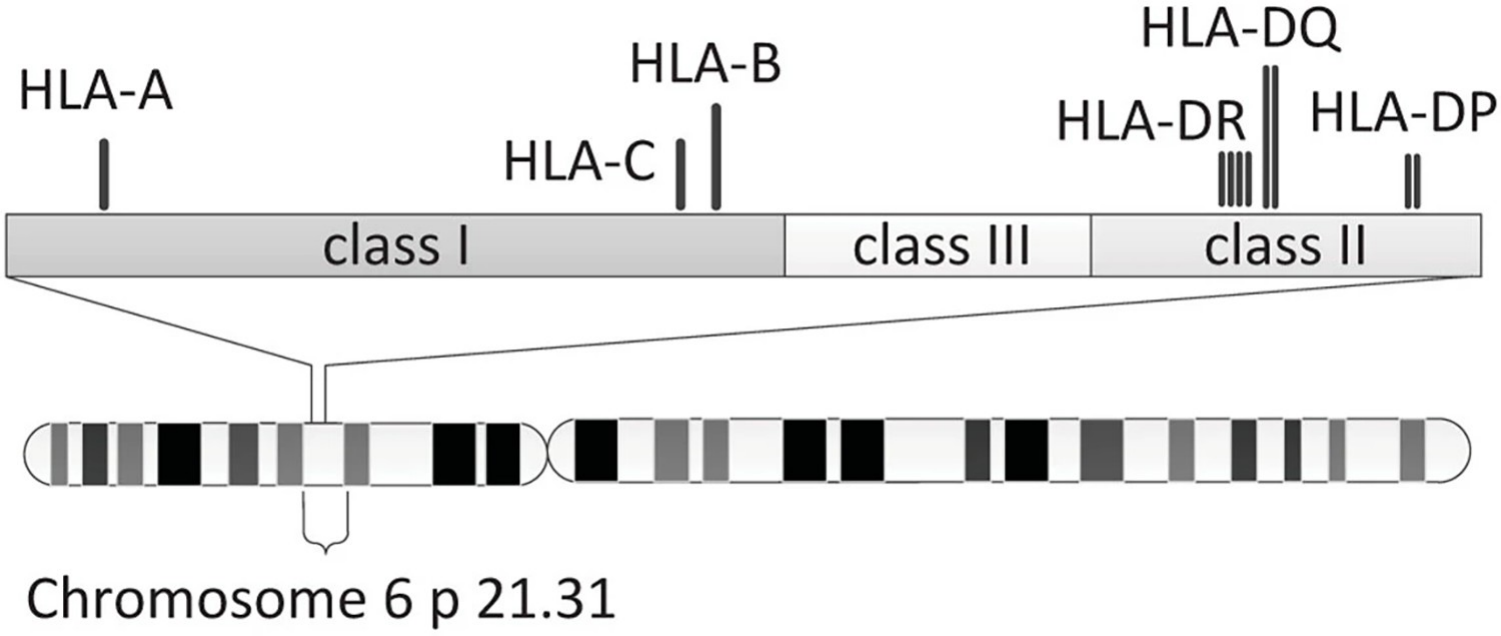
Diagrammatic representation of the HLA region [3].

The HLA region is classified into three main classes: I, II, and III (Fig 1) [3]. Class I comprises HLA-A, HLA-B, and HLA-C genes that encode the heavy chains of class I molecules. Class II consists of HLA-DR, HLA-DQ, and HLA-DP subregions, each containing A and B genes encoding α and β chains, respectively [4]. Class III encodes several molecules important in inflammation, such as complement components C2, C4, and factor B, Tumor Necrosis Factor-alpha, lymphotoxin, and three heat shock proteins [5].

The HLA region plays an important role in the innate and adaptive immune system [6], the complement cascade system [5], cord blood, and bone marrow transplants [7]. Specific HLA proteins have been associated with cancer development [8], a wide range of autoimmune and infectious diseases [9], and adverse drug reactions [10]. Identifying the exact HLA alleles associated with diseases is paramount to discovering the underlying genetic pathophysiology [11] and potential therapeutic targets.

HLA imputation infers an individual’s HLA genotype using SNP genotype information at sites flanking the classical HLA loci [12]. Prior to imputation, microarrays are used to collect SNP data from many samples at a moderately low cost. HLA alleles are then statistically imputed considering the long-range disequilibrium between the HLA loci and SNP markers across the HLA region, as described by Leslie *et al*., (2008) [13–15]. Imputation is a cheaper alternative to lab-based HLA typing, made possible due to the availability of large SNP datasets [12]. Imputation, combined with a larger database of reference haplotypes, can enable large-scale investigations, such as disease-association studies [16], where precise knowledge of the HLA type is essential.

Available HLA imputation tools use different algorithms. For instance, HIBAG uses attribute BAGging to maximize the advantages of bootstrap aggregation and random variables selection methods to improve accuracy [17]. SNP2HLA and CookHLA use BEAGLE [18] ⁠to impute HLA alleles and amino acid sequences, while Minimac4 uses the MaCH algorithm [19].

African genomes are more diverse and have a reduced linkage disequilibrium, making it even more challenging to impute HLA alleles [20]. Africa is regarded as the cradle of modern humans, *Homo sapiens*. Populations on other continents descended from groups that migrated from Africa thousands of years ago [21]. Genome-wide SNP genotyping revealed that African populations have maintained a large and subdivided structure throughout evolutionary history [22], and that the deepest splits between human populations lie in Sub-Saharan Africa [23, 24].

Assessing imputation accuracy is necessary as it is based on statistical inferences which involve probabilities. Additionally, the HLA region is highly variable as the alleles are inherited in a Mendelian fashion from each parent and thus vary from individual to individual [25]. Imputation performance can be affected by genotyping arrays, the number of individuals in the reference panel, the genetic and ethnic diversity represented, data quality, statistical method of the imputation tools, and how well the reference and study panels match.

Most studies that have assessed HLA imputation accuracy have used European, Asian, or multi-ethnic population data [10, 26, 27]. Previous studies have focused on evaluating general rather than HLA imputation accuracy in African populations [28]. The few studies that have examined HLA imputation accuracy in African populations have used target datasets from African Americans [29]. This study aimed to evaluate HLA allele imputation accuracy in a West African population, which has not been extensively studied, despite the heaviest disease burden occurring in Africa [30].

The study used GGVP data typed using the Optitype tool [31] as the gold standard to assess the performance of 4 imputation tools, three HLA-specific and one general. In addition, we also tested the effect of a population-specific versus a non-population-specific reference panel on imputation in a West African population. Finally, we assessed the impact of using data genotyped on different platforms and reference sample sizes for HLA imputation.

These results inform future GWAS studies on the most appropriate software, recommend reference panels for HLA imputation, and highlight the influence of genotyping arrays and reference panel size on HLA imputation accuracy.

## Materials and methods

### Study populations

The study used reference panels from the 1000 Genomes (1kg-All), 1000 Genomes African (1kg-Afr), 1000 Genomes Gambian (1kg-Gwd), Human Hereditary and Health in Africa (H3Africa), and the HLA Multi-ethnic datasets.

The study Whole Genome Sequence (WGS) dataset was derived from the Gambian Genome Variation Project (GGVP), a collaborative project between MRC Unit in the Gambia, the Wellcome Sanger Institute, and the MRC Centre for Genomics and Global Health at Oxford University. The GGVP dataset, supports the discovery and understanding of genetic variants influencing human diseases [32]. The GGVP datasets are open-access and can be found on the International Genome Sample Resource site [33]. Table 1 provides the sample size, number of SNPs, and number of HLA alleles for each dataset, while Table 2 describes the number of SNPs for each HLA locus across all datasets.

**Table 1:** List of the study target datasets and reference panel populations.

	Study populations	Sample size	Number of MHC SNPs	Number of HLA alleles
**Target dataset**				
Gambian individuals from GGVP WGS [32]	Fula, Jola, Wolof, Mandinka	315	**H3Africa array** 13,436 **Omni array** 13,850	
**Reference Panels**				
1kg-All	All 1000 Genomes populations [34].	2,051	223,529	425
1kg-Gwd	Gambian subpopulation within the 1000 Genomes [34].	95	223,529	139
1kg-Afr	African subpopulation within the 1000 genomes [34].	547	223,529	264
H3Africa	African datasets	1,089	187,222	281
HLA Multi-ethnic [35]	Japan Biological Informatics Consortium [36], the BioBank Japan Project [37], the Estonian Biobank [38], the 1000 Genomes Project [34], and a subset of studies in the TOPMed program [39].	21,546	-	-

**Table 2:** Number of SNPs for each HLA locus across datasets.

HLA locus	Dataset	Number of SNPs
HLA-A	Illumina Omni 2.5 array	62
	H3Africa array	47
	1kg-All reference	370
	H3Africa reference	592
	1kg-Gwd	370
	1kg-Afr	370
HLA-B	Illumina Omni 2.5 array	46
	H3Africa array	29
	1kg-All reference	587
	H3Africa reference	650
	1kg-Gwd	587
	1kg-Afr	587
HLA-C	Illumina Omni 2.5 array	49
	H3Africa array	35
	1kg-All reference	258
	H3Africa reference	299
	1kg-Gwd	258
	1kg-Afr	258

We used the Illumina Omni 2.5 and the H3Africa array marker sets [40] to assess how the density of markers on the target dataset could affect the imputation performance of HLA alleles. The H3Africa array is based on the Illumina Omni 2.5 array, with approximately 75% markers overlapping with the Illumina Omni array, and the remaining 25% markers being custom-made. The Illumina Omni 2.5 array and the H3Africa array target datasets were created by selecting matching markers from the GGVP WGS datasets and masking the remaining SNPs.

### HLA imputation strategy

The study focused on HLA class I alleles, the only class typed by OptiType [31]. Four tools were used to impute HLA alleles. These included HLA allele specific imputation tools HIBAG version 1.14.0 [41], CookHLA [42], SNP2HLA [14], and a general imputation tool, Minimac4 [43]. For SNP2HLA, PLINK version 1.07 was used for quality control, while BEAGLE version 3.0.4 was used for phasing and imputation. The Optitype [31] tool in the nf-core HLA typing pipeline [44, 45] was used to type HLA alleles. We then used Python scripts to combine HLA types into the required format for HIBAG, CookHLA, and SNP2HLA.

We used HLA Multi-ethnic [35] ready-made reference panel and customized four others—1kg-All, 1kg-Afr, 1kg-Gwd, and H3Africa—using HLA types and SNP genotypes for each imputation tool. For CookHLA, we fist generated a genetic map using the “MakeGeneticMap” module, then trained the reference panel using “MakeReference” module. The “MakeReference” module and the “hlaAttrBagging” function were used to train the SNP2HLA and HIBAG specific reference panels, respectively. For Minimac4, reference panels were generated using SNP genotypes and HLA alleles typed using the HLA-LA tool [46] instead of OptiType, matching the method used to create the HLA Multi-ethnic reference panel and thus enabling comparison.

HLA alleles were then imputed from SNP data using the “SNP2HLA” script with window size set to the default of 1000 for SNP2HLA and the “hlaPredict” function for HIBAG. For CookHLA, the “CookHLA.py” script was used for imputation. For Minimac4, HLA alleles were imputed by calling the Minimac4 tool. For the HLA Multi-ethnic reference panel, the sample datasets were submitted to the Michigan imputation server [47], and HLA imputation was conducted using the Minimac4 imputation tool.

### Imputation accuracy assessment

We used concordance rate as the primary assessment metric, which is the percentage of correctly imputed best-guess alleles of all imputed alleles based on true HLA alleles. The true HLA alleles were obtained by typing HLA alleles from GGVP WGS data using OptiType tool, which has been shown to type HLA Class I alleles at 99% accuracy [48]. The “hlaCompareAllele” function in HIBAG was used to calculate the concordance rate, while the “measureacc” module in the CookHLA package [42] was used to calculate the SNP2HLA, CookHLA, and Minimac4 concordance rate.

The accuracy of results can also be assessed using HLA allele error rates. HLA allele frequency, which reflects the genetic diversity in a population, can also evaluate the accuracy of HLA alleles. HLA allele frequencies were computed using the PyPop [49] package and compared with concordance rates.

### Reproducibility

For reproducibility, we automated the pipeline in the Nextflow workflow language, packaged and deployed the tools using Docker and Singularity containers, and used GitHub for documentation and version control [50].

**Fig 2:**
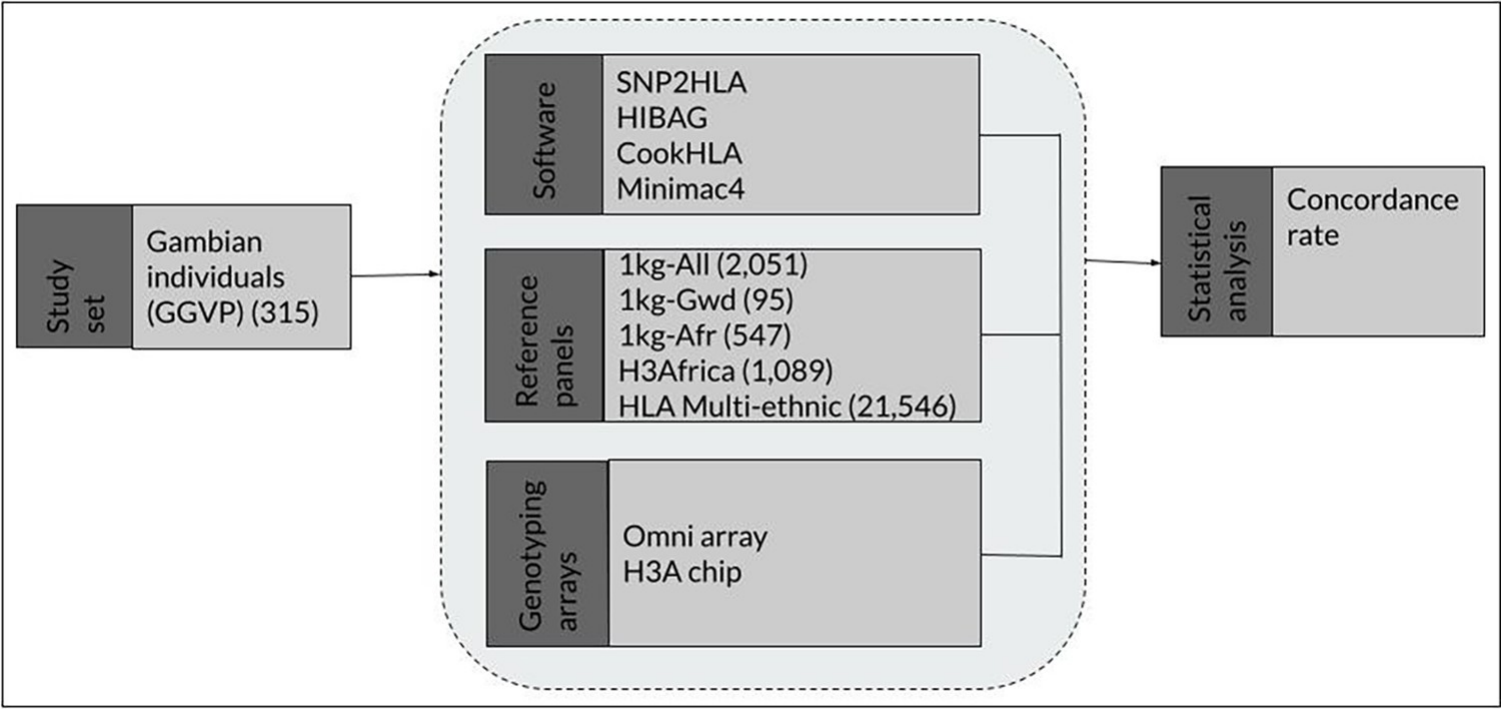
A summary of the materials and methods used.

A summary of the workflow used for the analysis is presented in Fig 2. Matching markers from the GGVP WGS datasets were chosen to produce the target datasets for the Illumina Omni 2.5 and the H3Africa arrays. The datasets were then imputed on 5 reference panels using 4 imputation tools, and HLA imputation accuracy was assessed using concordance rate.

## Results

### Sample data

The target dataset was obtained from the GGVP WGS dataset and used to select matching markers on the H3Africa and Illumina Omni 2.5 arrays. Of the 1,731,033 SNP markers on the H3Africa array, 13,436 MHC SNPs matched those in the GGVP WGS dataset, while 1,717,596 were unique to the H3Africa array. Of the 2,314,963 SNP markers on the Illumina Omni 2.5 array, 13,850 MHC SNPs matched those in the GGVP WGS dataset, while 2,301,113 were unique to the Illumina Omni 2.5 array. The 13,436 H3Africa array SNPs and 13,850 Illumina Omni 2.5 array SNPs were used as the sample datasets.

Table 3 describes the intersection between markers in the reference panel compared to the target array data. For example, of the 223,229 markers in the 1kg-All reference, 13,016 matched those in the Illumina Omni 2.5 array, while 210,213 were unique to the 1kg-All reference.

**Table 3:** Intersection of target datasets with reference datasets.

Target dataset	Reference	Matching markers	Nonmatching markers
Illumina Omni 2.5 array	1kg-All	13,016	210,213
	H3Africa	13,038	174,184
	1kg-Gwd	13,016	210,213
	1kg-Afr	13,016	210,213
H3Africa array	1kg-All	13,098	210,431
	H3Africa	12,865	174,357
	1kg-Gwd	13,098	210,431
	1kg-Afr	13,098	210,431

### Imputation concordance

Table 4 shows the concordance rate for the different imputation tools, genotyping arrays, and reference panels. Compared to HLA typing, the overall concordance rate of the imputed data was 0.837 for HIBAG, 0.769 for Minimac4, 0.584 for SNP2HLA, and 0.173 for CookHLA. The HLA Multi-ethnic was the best performing reference panel with an accuracy rate of 0.873, followed by the H3Africa panel at 0.619, then 0.609 for 1kg-Afr, 0.604 for 1kg-All and 0.531 for 1kg-Gwd. For the array comparison, data from the Omni 2.5 was more accurate than data from H3Africa. The Omni 2.5 array contained a few more Gambian SNPs than the H3Africa array, which would likely impact results. The averages exclude the HLA Multi-ethnic reference panel due to missing values.

**Table 4:** Overall concordance rate.

	Reference panels					
	1kg-All	1kg-Afr	1kg-Gwd	H3Africa	HLA Multi-ethnic	
Imputation program						Average
SNP2HLA	**0.656**	0.64	0.425	0.614	**-**	0.584
HIBAG	0.830	0.850	0.779	**0.889**	**-**	0.837
CookHLA	0.101	0.172	0.208	**0.212**	**-**	0.173
Minimac4	0.83	0.777	0.711	0.761	**0.873**	0.769
**Average**	0.604	0.610	0.531	0.619	0.873	

There was no comparison of SNP2HLA, HIBAG, and CookHLA on the HLA Multi-ethnic panel, as the Michigan imputation server that contains the reference panel was prebuilt with Minimac4 only. From the analysis, HLA-C (0.668) allele imputation was found to be most accurate, followed closely by HLA-A (0.618) and lastly, HLA -B (0.551), as shown in Table 5.

**Table 5:** Allele-specific concordance rate.

			Reference Panels				
			1kg-All	1kg-Afr	1kg-Gwd	H3Africa	HLA Multi-ethnic [22]
Genotyping Array	Allele	Imputation program	Concordance Rate				
Omni array	HLA-A	SNP2HLA	0.749	**0.759**	0.408	0.725	-
		HIBAG	0.877	0.923	0.839	**0.926**	-
		CookHLA	0.035	0.098	**0.219**	0.147	-
		Minimac4	0.760	0.649	0.541	0.682	**0.914**
	HLA-B	SNP2HLA	**0.598**	0.557	0.381	0.551	-
		HIBAG	0.737	0.728	0.699	**0.808**	-
		CookHLA	0.094	0.224	0.203	**0.240**	-
		Minimac4	0.840	0.805	0.784	0.689	**0.854**
	HLA-C	SNP2HLA	**0.616**	0.584	0.463	0.611	-
		HIBAG	0.941	0.947	0.847	**0.969**	-
		CookHLA	0.187	0.268	**0.294**	0.281	-
		Minimac4	0.868	0.865	0.849	**0.905**	0.865
H3Africa array	HLA-A	SNP2HLA	0.771	**0.775**	0.517	0.710	-
		HIBAG	0.885	0.915	0.818	**0.929**	-
		CookHLA	0.078	0.063	0.219	**0.238**	-
		Minimac4	0.868	0.752	0.570	0.732	**0.917**
	HLA-B	SNP2HLA	**0.579**	0.535	0.335	0.522	-
		HIBAG	0.640	0.680	0.651	**0.754**	-
		CookHLA	0.073	**0.170**	**0.170**	0.149	-
		Minimac4	0.775	0.737	0.687	0.668	**0.825**
	HLA-C	SNP2HLA	**0.625**	**0.625**	0.446	0.570	-
		HIBAG	0.900	0.908	0.819	**0.952**	-
		CookHLA	0.138	0.210	0.141	**0.216**	-
		Minimac4	0.852	0.851	0.835	**0.887**	0.863

### Imputation accuracy based on reference panels

**Fig 3:**
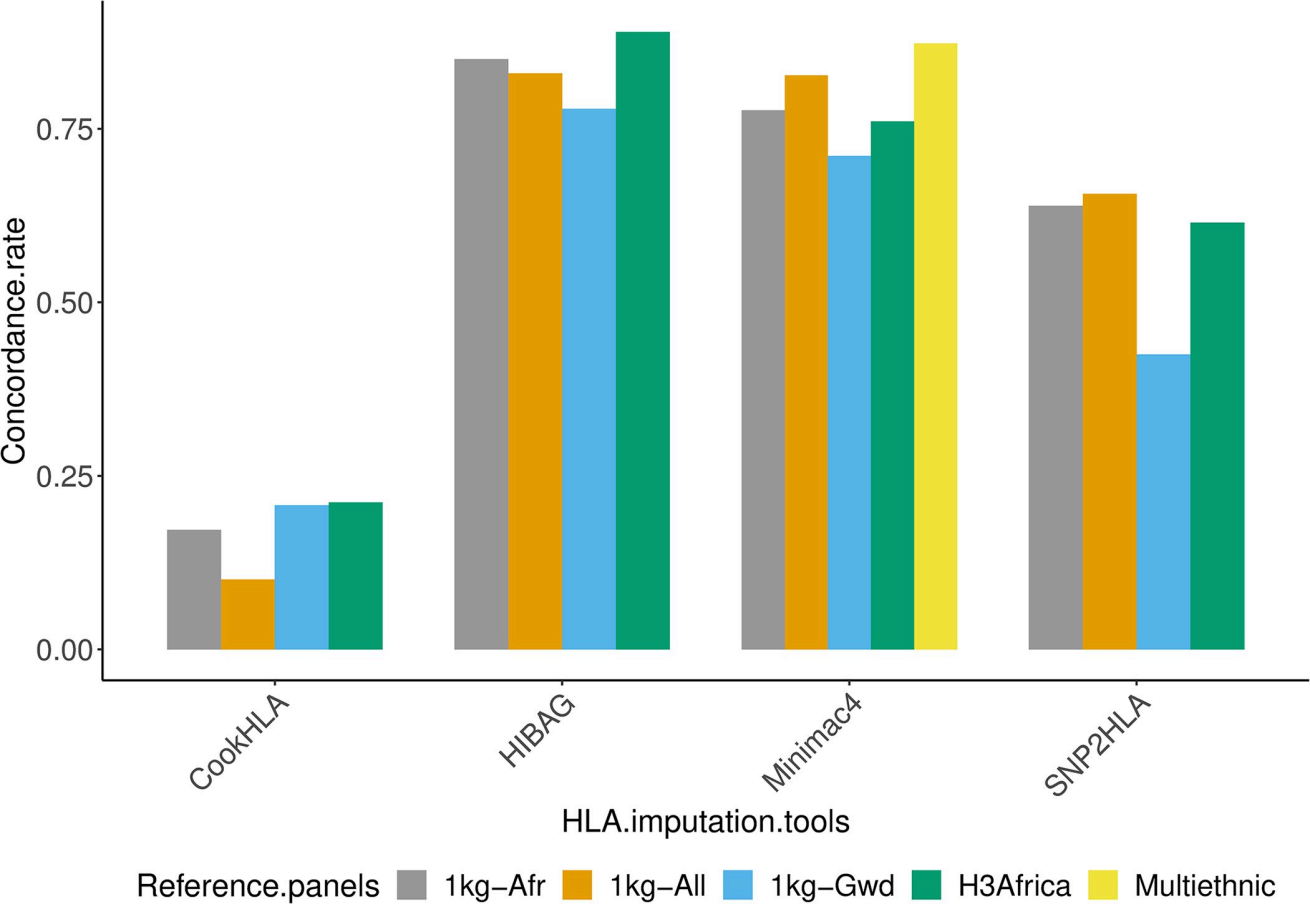
Concordance rate based on reference panels.

The H3Africa reference panel had the highest concordance with HLA typing using HIBAG (0.889) and CookHLA (0.212). The 1kg-All was the best performing reference panel for SNP2HLA (0.656), while the HLA Multi-ethnic had the highest concordance rate when using Minimac4 (0.873) (Fig 3).

### Comparison of allele frequency and accuracy of HIBAG

**Fig 4:**
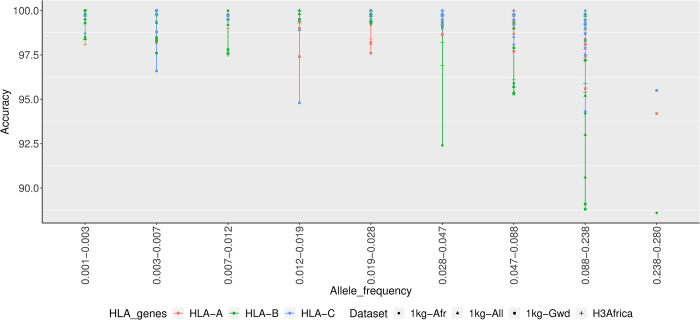
Allele frequency vs. accuracy of HIBAG. Accuracy tended to decrease with increasing frequency, especially for HLA-B alleles.

HLA alleles imputed by HIBAG, the best performing imputation tool, were used for allele frequency and accuracy rate comparison (Fig 4). HLA imputation accuracy dropped when the frequency of HLA alleles increased across all the reference panels, especially for the HLA-B alleles.

### Imputation accuracy based on error rates

**Fig 5:**
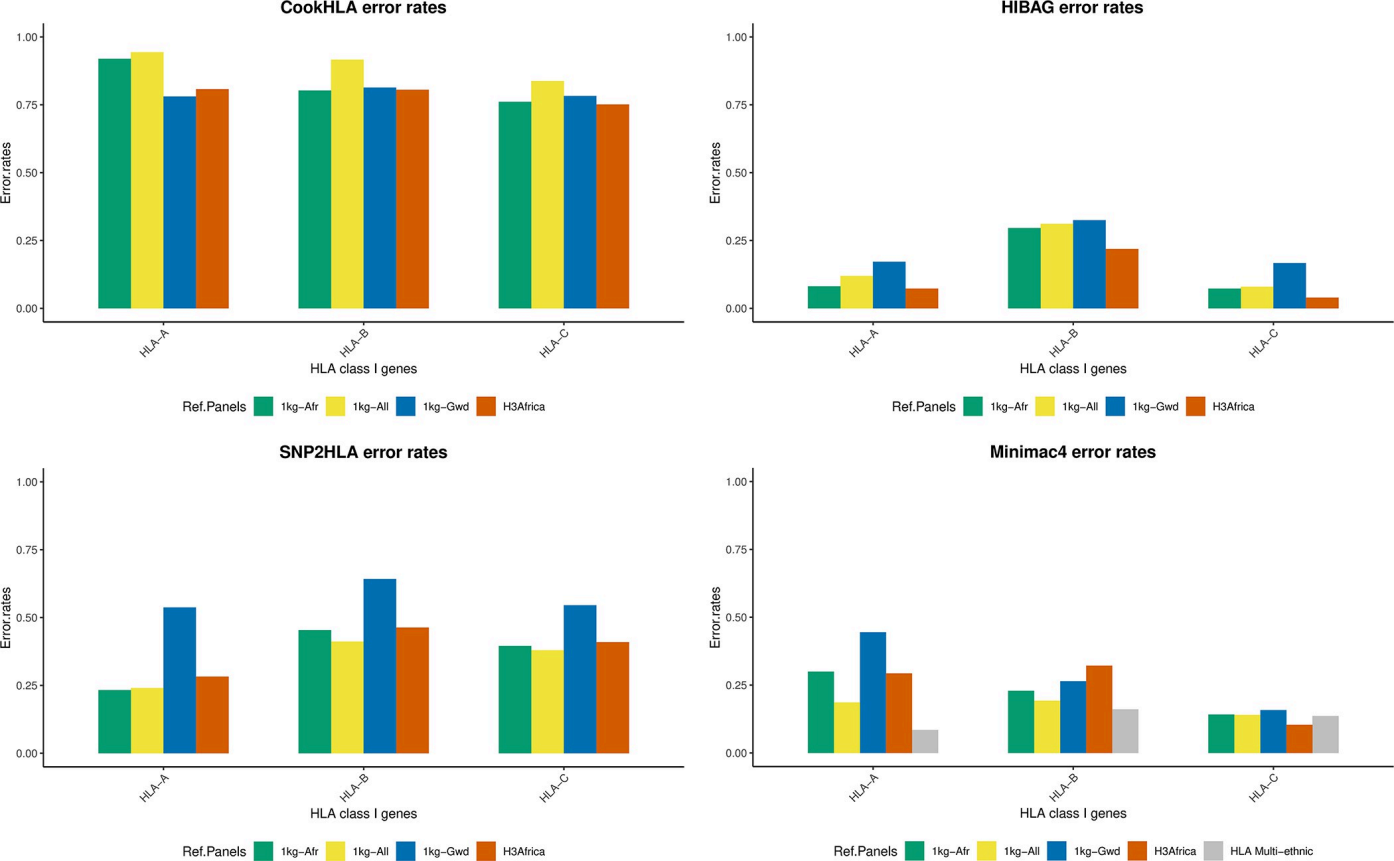
Imputation accuracy comparison based on error rates. Results from HIBAG (Fig 5B) showed that HLA-B had a higher error rate, followed by HLA-A and HLA-C. HLA-B imputation was less accurate for SNP2HLA (Fig 5C), followed by HLA-C and HLA-A. HLA-A had higher error rates for Minimac4 (Fig 5D) and CookHLA (Fig 5A), followed by HLA-B and HLA-C alleles.

Overall, HLA-B alleles had higher error rates (0.449) compared to HLA-A (0.382) and HLA-C (0.332), showing they were imputed less accurately. CookHLA imputed HLA alleles with the highest error rates (Fig 5A). An interesting observation was that Minimac4, a general imputation tool, imputed HLA-B alleles more accurately than any HLA-specific imputation tool.

## Discussion

We provide a detailed comparison of five reference panels, four imputation tools, and two genotyping arrays used for HLA imputation in a West African population. HIBAG and the H3Africa reference panel were the best performing imputation tool and reference panel, respectively.

The high performance of HIBAG is expected, as shown in a previous study [51]. Furthermore, HIBAG is robust for populations with complex linkage disequilibrium blocks [10]. Compared to Minimac4, SNP2HLA, and CookHLA, HIBAG uses unphased genotyped data, eliminating variation provided by phasing software and shortening the computational phasing steps. Regarding computational burden, HIBAG takes a long time to run when the reference panel needs to be customized. For instance, the 1kg-All reference panel, which was the largest, took approximately 20 days and 32 threads when training with HIBAG compared to a few hours with 9 threads when training with SNP2HLA. SNP2HLA provides an added advantage over HIBAG, since it imputes HLA SNPs, amino acids, and alleles, unlike HIBAG, which imputes only HLA alleles.

In general, the H3Africa reference panel outperformed the other reference panels due to its larger sample size and its relationship to the target population. Generally, the size of the reference panel [18] and the population specificity [51] substantially affect the accuracy of the HLA allele imputation. As expected, increased accuracy was achieved with a more extensive HLA Multi-ethnic reference panel, but we could not compare it with other tools as the server only provides the Minimac4 tool. Specifically, the H3Africa panel outperformed the other panels when using HIBAG, while the 1kg-All reference performed better with SNP2HLA, implying that the performance of HIBAG was based on population specificity and sample size [51], while the performance of SNP2HLA was based only on sample size [14]. The decrease in HLA imputation accuracy with increased frequency is comparable to a study by Karnes *et al*. (2017), who demonstrated that most low frequency HLA alleles had high concordance rates in African Americans and European Americans [26].

The performance of the Illumina Omni 2.5 array was slightly better than that of the H3Africa array because it has more SNPs in the target population, 13,850 SNPs, compared to 13,436 SNPs. However, this difference was statistically insignificant, showing that the choice of genotyping arrays has little influence on the accuracy of HLA imputation. However, note that the two arrays have significant overlap in their content, which may explain the similarities, therefore, it is necessary to compare more diverse arrays to fully assess the impact of array content. Verlouw *et al*. (2021) showed that genome-wide coverage of genotyping arrays correlates with the number of SNPs in genotyping arrays but does not correlate with the imputation quality [52]. Therefore, the choice of genotyping arrays should be based on additional genotyping array content, such as pharmacogenetics or HLA variants, and not only on the extent of genome coverage of genotyping arrays.

Imputation of HLA-B (0.551) was less accurate compared to HLA-A (0.618) and HLA-C (0.668) imputation. Accurately typing alleles in the HLA-B region is problematic due to high polymorphism [53]. According to Robinson *et al*. (2015), over 3000 allelic variants exist in the HLA-B region [54]. However, accurate imputation of HLA-B alleles is important, as they play a crucial role in the progression of acquired immune deficiency syndrome. A slow progression of the disease has been associated with individuals expressing HLA-B*57 and HLA-B*27, while rapid progression has been associated with individuals expressing HLA-B*35 alleles [55]. Minimac4 showed improved imputation accuracy of HLA-B alleles, suggesting that a general imputation tool can be used for studies targeting HLA-B alleles.

## Conclusions

The most effective software for HLA allele imputation in this study was HIBAG. However, it has a long run time and high memory requirement during the training of the reference panel. A recommendation is to use HIBAG with the latest kernel version, 1.5, as it has GPU support. Another important observation is that reference panel sample size and population content influence HLA allele imputation accuracy.

This study identified factors to consider when selecting an imputation tool and reference panel to inform association studies focusing on the HLA region and West African populations. The results highlight the best tools and panels for accurately imputing HLA genotypes.

We recommend testing additional African populations other than the Gambian population to better assess imputation accuracy in specific African populations. Such an assessment was done recently for imputation across the genome [56], and we encourage more studies especially within the HLA region. Reference panels comparable in size should be used to reduce bias, where a single large panel outperforms smaller ones.

Building large African-specific reference panels will enable high-quality imputations, especially for studies that cannot afford the cost of next-generation sequencing, thus generating more data that can be used for genome-wide association and fine-mapping studies in African populations.
